# Some of the Cheap Preparations of the Bark—Cinchonia Alkaloid—Dextro-Quinine

**Published:** 1880-01-20

**Authors:** T. B. Greenley

**Affiliations:** Kentucky


### *SOME OF THE CHEAP PREPARATIONS OF THE BARK.*

BY T. B. GREENLEY, OF KENTUCKY.

### CINCHONIA ALKALOID.

Within the past twelve months I have used several cheap preparations of the bark, viz.: Cinchonia alkaloid, both plain and prepared; and also what is called dextro-quinine. The former of these preparations is from the house of Messrs. Powers & Weightman, and the latter from Messrs. Keasby & Mattison, both of Philadelphia.

Up to the first of January last I had reported some twenty or more cases of intermittent and remittent fever in which I had used the alkaloid preparations successfully as a substitute for quinine. Since that time I have treated as many more cases with equal success. These cases were mostly intermittent, but I found that they answered also very well in the remittent type of malarial fever.

They are extremely well adapted to children or even others where there is great intolerance of medicines on account of disgust of the stomach for everything in that way. They can be rubbed down with loaf sugar or mixed with cream, and given to a child when it will not be conscious of taking medicine. The same may be said of other patients where great disgust for medicine exists. It is a hard matter to mix these medicines with water.

I have found that it is always best to allow the patient to have acidulated drinks while under treatment. The dose of the plain alkaloid is much smaller than the prepared, being about half the bulk of the sulphate of quinine. The prepared alkaloid consists of 12 parts the medicine to 60 parts of sugar of milk, and 1 part bicarb. soda; and consequently makes the bulk much larger, and renders it necessary to give about four times as much at a dose.

These medicines are entirely exempt from the bitter taste that naturally belongs to quinine and other preparations of the bark. This want of taste in that respect is due to their insolubility in the mouth; hence the necessity of the use of acidulous drinks during their exhibition to aid their solution in the stomach. We occasionally find a patient with sour stomach who may not need the acid drinks. The acidulated drinks may consist of either lemonade, sometimes vinegar and water, hard cider or butter milk, all of which will aid the solution.

### DEXTRO-QUININE.

I find the dextro-quinine a most admirable preparation for the treatment of intermittent fever. Besides it anti-periodic effect, it seems to possess a bracing, tonic property which is well adapted to the treatment of that variety of malarial trouble.

This medicine is prepared from chinoidine, a principle left as a residuum in the preparation of the salts of the bark, and will not crystalize; consequently contains more or less of all the elements of the bark.

The dose in bulk is not so large as that of sulphate of quinine, as it is heavier.

The cost of these medicines is not half as great as that of quinine, which is quite an item to be considered with poor people, as well as with most country doctors, who in many instances have to furnish medicines without compensation.

I apprehend they will soon attain great reputation, both among the profession and the people.

Thanks to the proprietors for samples of these preparations.

